# Supplementary material for: Identification of structural and regulatory cell-shape determinants in Haloferax volcanii
Source: Nat Commun. 2024 Feb 15;15:1414. doi: 10.1038/s41467-024-45196-0 (PMC10869688; doi:10.1038/s41467-024-45196-0)
Supplement: Supplementary file 1 — Supplementary Information [file 41467_2024_45196_MOESM1_ESM.pdf]

Supplementary Information for

Identification of structural and regulatory cell-shape determinants in *Haloferax volcanii*

Heather Schiller[1], Yirui Hong[1], Joshua Kouassi[1], Theopi Rados[2], Jasmin Kwak[2], Anthony DiLucido[1], Daniel Safer[3], Anita Marchfelder[4], Friedhelm Pfeiffer[4,5], Alexandre Bisson[2*], Stefan Schulze[1,6*], and Mechthild Pohlschroder[1*]

[1] University of Pennsylvania, Department of Biology, Philadelphia, PA 19104, USA

[2] Brandeis University, Department of Biology, Waltham, MA 02453, USA

[3] University of Pennsylvania, Department of Physiology, Philadelphia, PA 19104, USA

[4] Biology II, Ulm University, 89069 Ulm, Germany

[5] Computational Biology Group, Max Planck Institute of Biochemistry, 82152 Martinsried, Germany

[6] Rochester Institute of Technology, Thomas H. Gosnell School of Life Sciences, Rochester, NY 14623, USA

[*]These authors jointly supervised this work

bisson@brandeis.edu

sxssbi1@rit.edu

pohlschr@sas.upenn.edu

This document includes:

Supplementary Figures 1 – 9, Supplementary Tables 1– 3, and Supplementary Notes 1 – 5

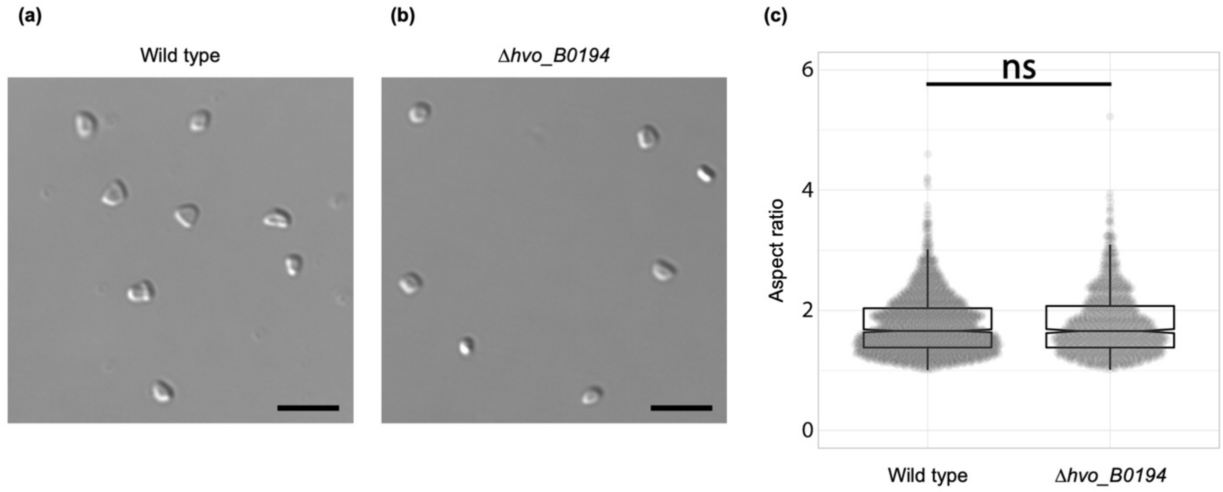

Supplementary Figure 1: Δhvo_B0194 *does not have a shape defect.* Late-log cell shape images for **(a)** wild type and **(b)** Δ*hvo_B0194* using DIC microscopy, each representative of three biological replicates, and imaged at OD_{600} between 1.55 to 1.65. Scale bars are 5 μm. **(c)** Quantification of aspect ratio for wild type and Δ*hvo_B0194* at late-log growth phase. n = 1683 and 1042 for wild type and Δ*hvo_B0194*, respectively. Aspect ratio comparisons were assessed using an unpaired, nonparametric, two-tailed Kolmogorov-Smirnov test. p = 0.2890. 'ns' is not significant. Aspect ratios <2 are considered disks and/or short rods. Boxplots show the mean (center line between boxes), interquartile range (boxes), 95% confidence interval of the mean (notch), as well as the lowest and highest 25th percentile of the distribution (whiskers). Source data are provided as a Source Data file.

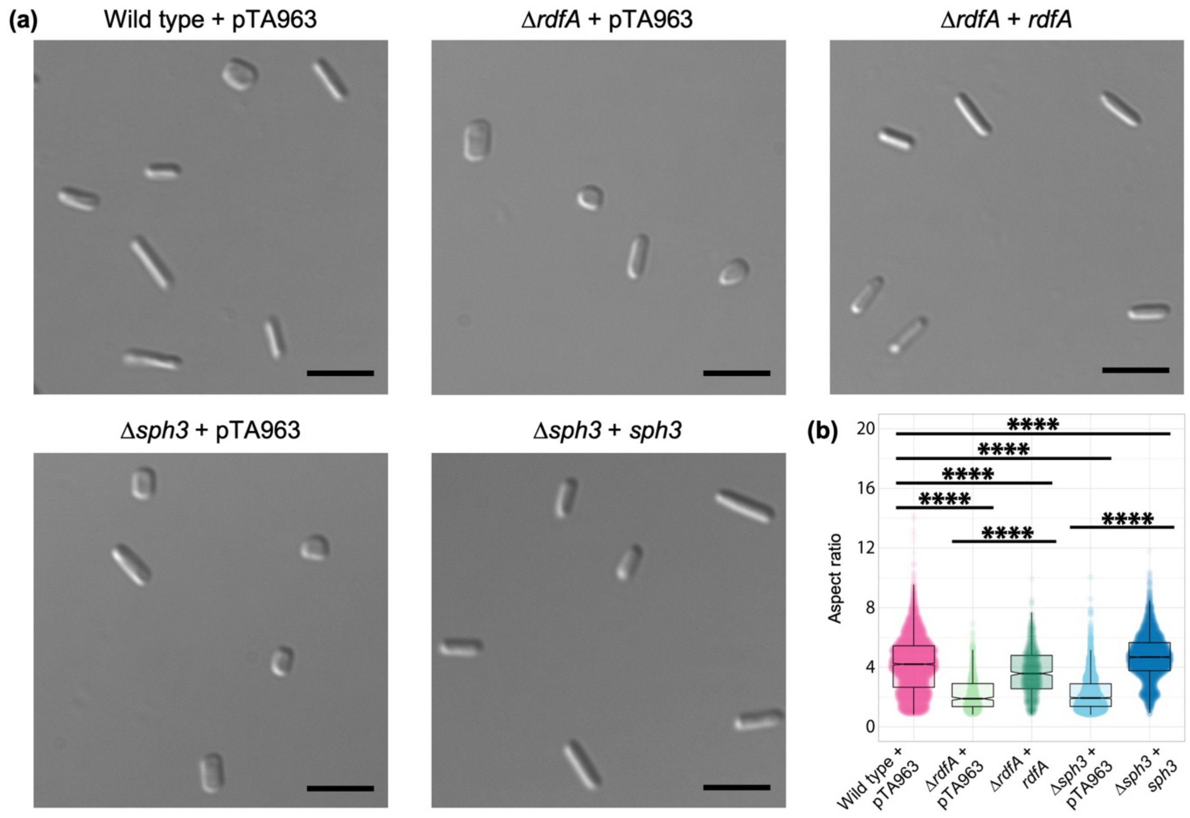

Supplementary Figure 2: Complementation of ΔrdfA *and* Δsph3. **(a)** Early-log (OD_{600} between 0.045 and 0.050) shape images, each representative of three biological replicates, for wild type + empty vector pTA963, Δ*rdfA* + empty vector pTA963, Δ*rdfA* + *rdfA*, Δ*sph3* + empty vector pTA963, and Δ*sph3* + *sph3*. Scale bars are 5 µm. **(b)** Quantification of cellular aspect ratio. n = 5028, 520, 758, 2129, and 4288 for wild type + empty vector, Δ*rdfA* + empty vector, Δ*rdfA* + *rdfA*, Δ*sph3* + empty vector, and Δ*sph3* + *sph3*, respectively. Aspect ratio comparisons were assessed using an unpaired, nonparametric, two-tailed Kolmogorov-Smirnov test. ****$p<0.0001$. Effect size is -0.798 between wild type + pTA963 and Δ*rdfA* + *rdfA*, 2.08 between Δ*rdfA* + pTA963 and Δ*rdfA* + *rdfA*, 0.591 between wild type + pTA963 and Δ*sph3* + *sph3*, and 3.419 between Δ*sph3* + pTA963 and Δ*sph3* + *sph3*. Aspect ratios <2 are considered disks and/or short rods. The presence of an empty vector has been shown to influence shape by increasing rod formation [1], which may

explain the presence of some rods in Δ*rdfA* + empty vector and Δ*sph3* + empty vector strains. Boxplots show the mean (center line between boxes), interquartile range (boxes), 95% confidence interval of the mean (notch), as well as the lowest and highest 25th percentile of the distribution (whiskers). Source data are provided as a Source Data file.

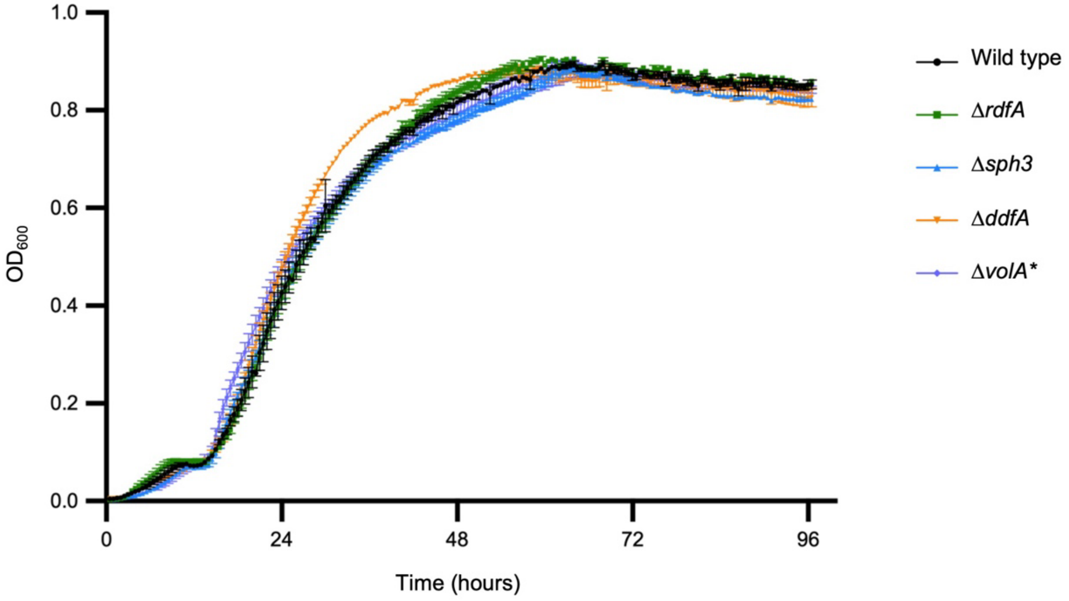

Supplementary Figure 3: Growth curve for wild type, ΔrdfA*,* Δsph3*,* ΔddfA*, and* ΔvolA*. Wild type and mutant strains were grown in a 96-well plate with double orbital shaking for approximately 96 hours, with OD_{600} readings taken every 30 minutes. Error bars are standard deviations across three biological replicates per strain. Source data are provided as a Source Data file.

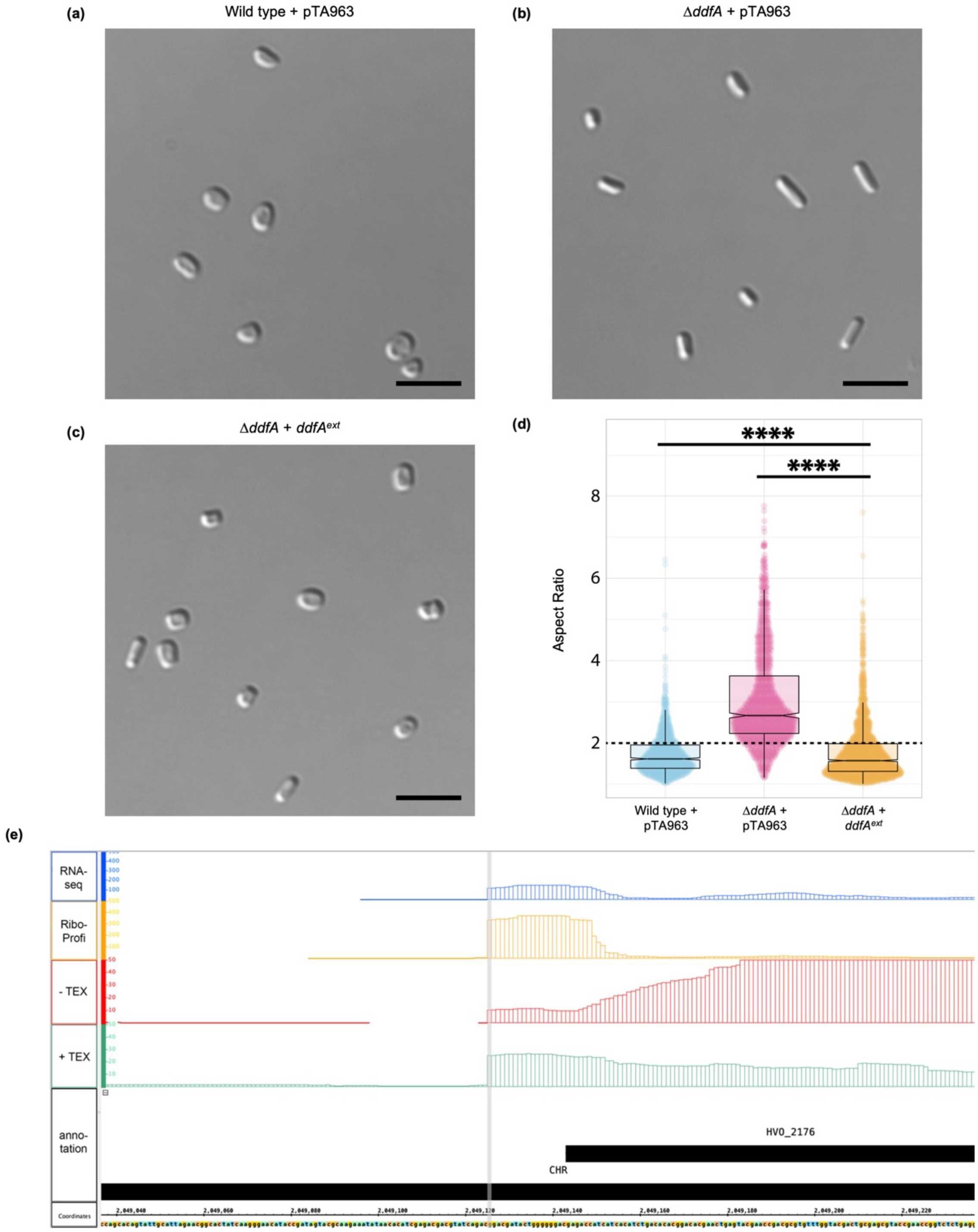

Supplementary Figure 4: Complementation of ΔddfA *and predicted start site of* ddfA. Late-log (OD_{600} between 1.7 and 1.8) shape images for **(a)** wild type + empty vector pTA963, **(b)** Δ*ddfA* + empty vector pTA963, and **(c)** Δ*ddfA* + *ddfA*^{ext}, each representative of three biological replicates.

Δ*ddfA* + *ddfA*^{ext} is the complementation strain; complementation was achieved using the re-annotated *ddfA* gene with an additional 126 nucleotides (*ddfA*^{ext}) added to the N-terminus along with a traditional ATG start codon. Scale bars are 5 μm. **(d)** Quantification of cellular aspect ratio. n = 1039, 1246, and 1972 for wild type + empty vector, Δ*ddfA* + empty vector, and Δ*ddfA* + *ddfA^{ext}*, respectively. Aspect ratio comparisons were assessed using an unpaired, nonparametric, two-tailed Kolmogorov-Smirnov test. ****$p<0.0001$. Effect size is -0.041 between wild type + pTA963 and Δ*ddfA* + *ddfA^{ext}* and -1.101 between Δ*ddfA* + pTA963 and Δ*ddfA* + *ddfA^{ext}*. Aspect ratios <2 are considered disks and/or short rods. Boxplots show the mean (center line between boxes), interquartile range (boxes), 95% confidence interval of the mean (notch), as well as the lowest and highest 25th percentile of the distribution (whiskers). **(e)** dRNA-Seq data [2] (green and red panels) reveal a promoter 18 base pairs upstream of the annotated *ddfA* (*hvo_2176*) gene. Red signals (panel -TEX) represent reads from an RNA fraction containing all cellular RNAs. Green signals (panel +TEX) represent reads from an RNA sample treated with terminator 5' phosphate-dependent exonuclease (TEX). Ribosome profiling data [3] are shown in orange (panel Ribo-Profi), and the corresponding RNA-seq data are shown in blue (panel RNA-seq). Ribosome profiling shows an enriched ribosome density in the 5' UTR, typical for a leadered mRNA, which is very likely generated by initiating ribosomes [3]. The genome coordinates and the annotation are shown at the bottom in black. Source data are provided as a Source Data file.

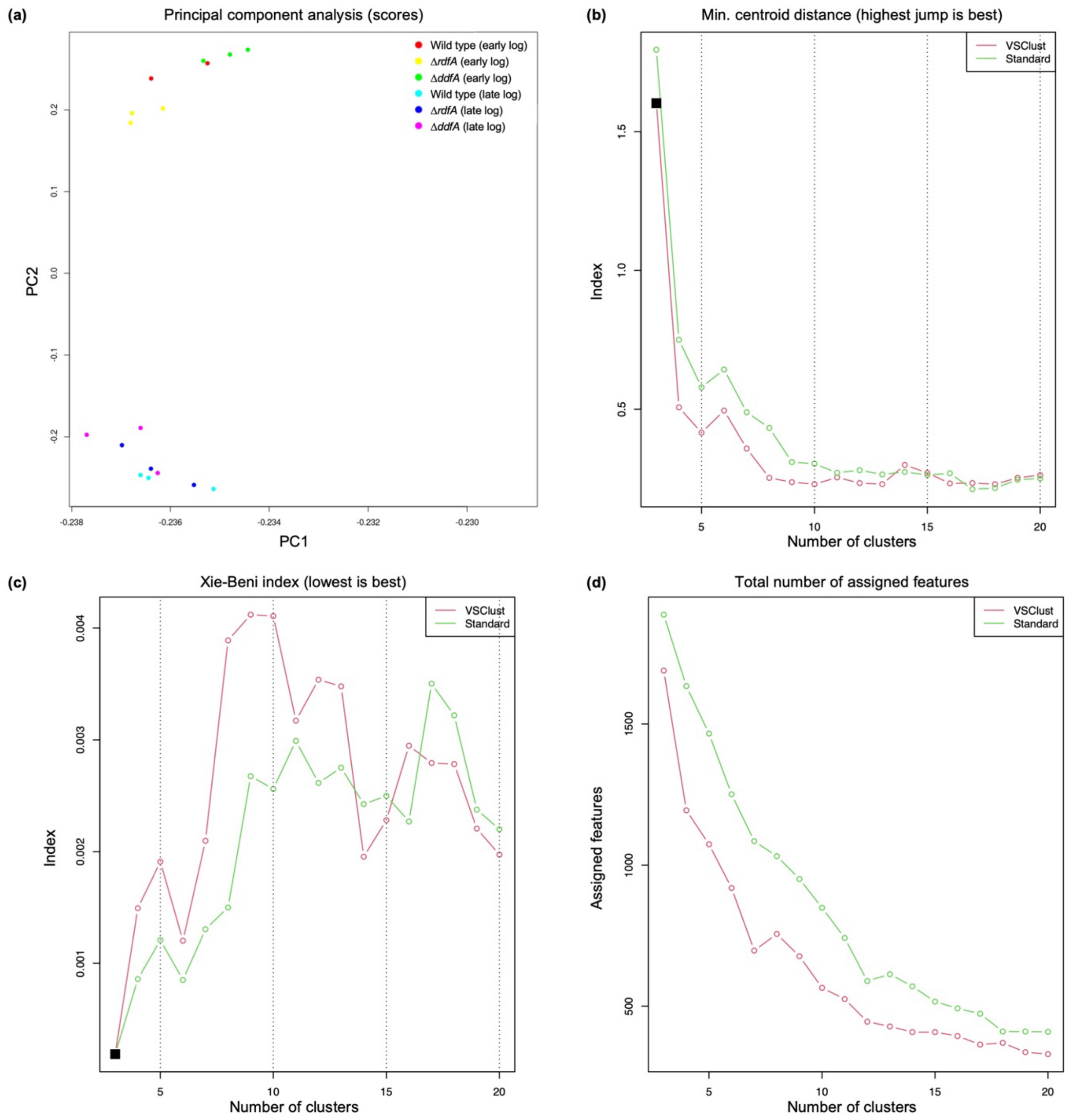

Supplementary Figure 5: Principal component analysis (PCA) and VSClust estimation of the number of variance-sensitive clusters. **(a)** PCA plot showing separation of strains and growth phases, with the largest separation observed for PC2, corresponding to differences in the growth phase. Conditions are labeled by color (inserted legend). VSClust reported three metrics for the estimation of cluster numbers: **(b)** Minimum centroid distance, **(c)** Xie-Beni index, and **(d)** total

number of assigned features. A cluster number of 14 was chosen because of the corresponding local maximum in (b) and local minimum in (c).

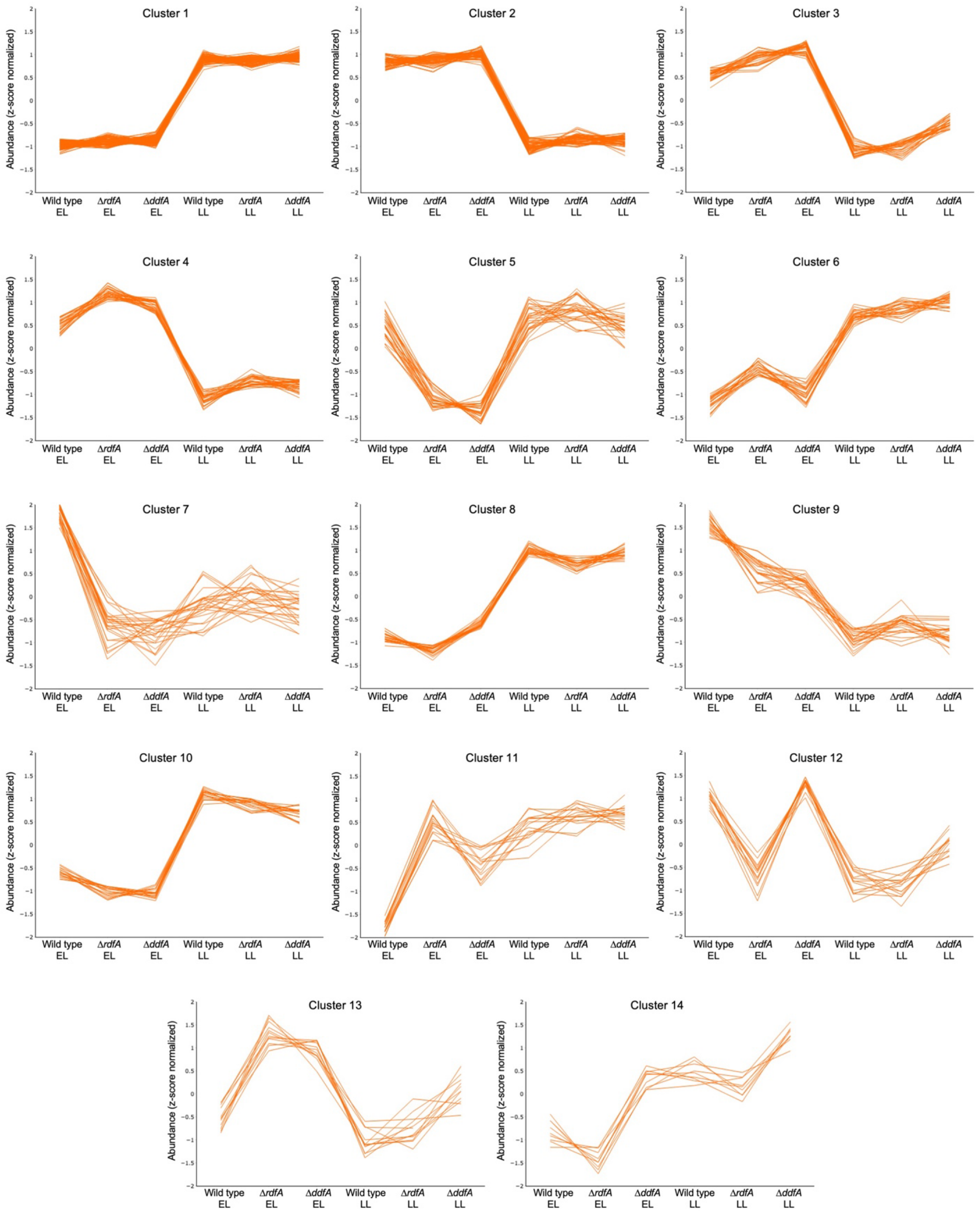

Supplementary Figure 6: Variance-sensitive clustering based on patterns of protein abundance across strains and growth phases, resulting in 14 clusters. Normalized protein abundances across

each condition are shown for each cluster, with individual proteins or protein groups represented as single lines. ‘EL’: early-log growth phase, ‘LL’: late-log growth phase. Raw data are provided in the Supplementary Data 2 file.

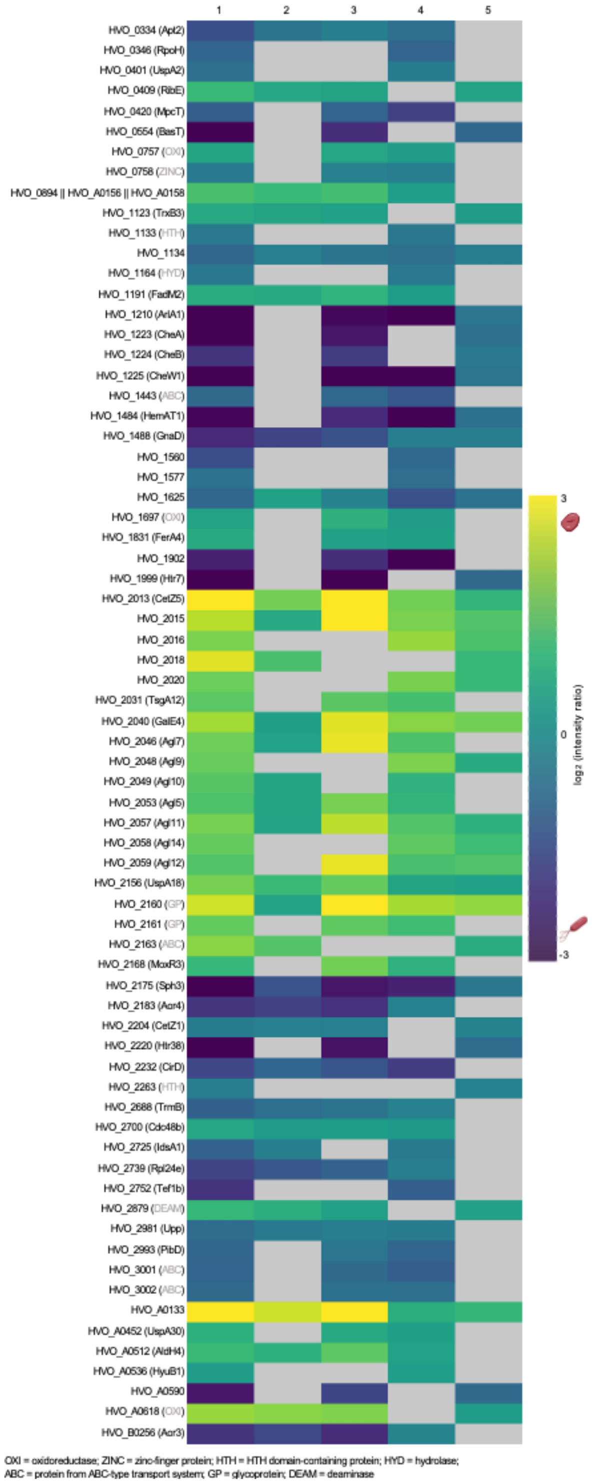

Supplementary Figure 7: Heatmap of proteins likely important for shape by proteomic comparisons between wild type, ΔrdfA*, and* ΔddfA. Proteins were filtered for protein abundance

ratios with a PEP<0.05 in column 1 and column 4 and/or 5; PEP>0.05 are gray boxes. Column numbers correspond to comparisons defined in Fig. 2a. Protein groups include names for all members, separated by ||. Raw data are provided in the Supplementary Data 3 file.

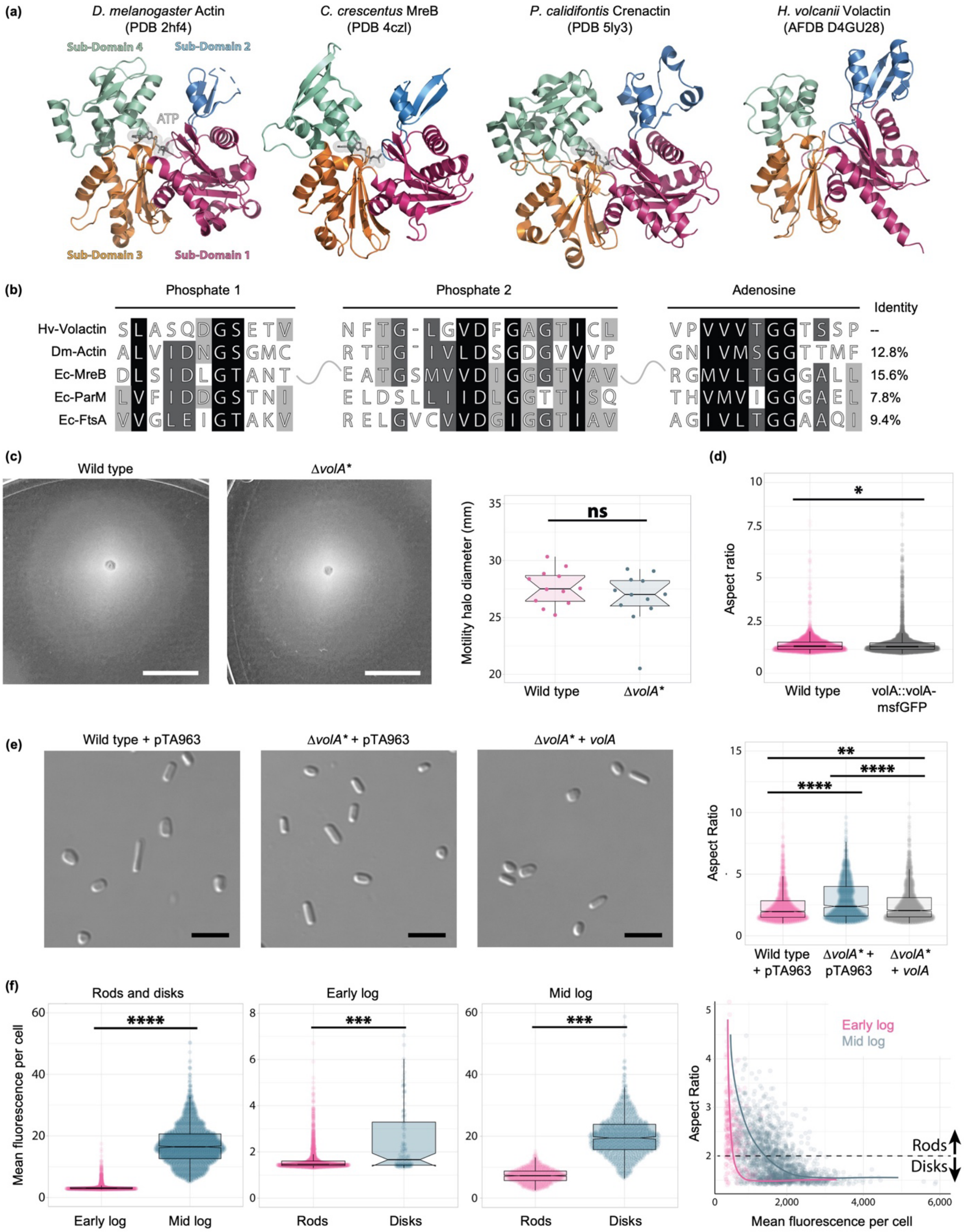
(a)
D. melanogaster Actin
(PDB 2hf4)
C. crescentus MreB
(PDB 4czl)
P. calidifontis Crenactin
(PDB 5ly3)
H. volcanii Volactin
(AFDB D4GU28)
Sub-Domain 4
Sub-Domain 2
ATP
Sub-Domain 3
Sub-Domain 1
(b)
Phosphate 1
Phosphate 2
Adenosine
Identity
Hv-Volactin SLASQDGSETV NFTG-LGVDFGAGTICL VPVVVTGGTSSP --
Dm-Actin ALVIDNGSGMC RTTG-IVLDSGDGVVVP GNIVMSGGTTMF 12.8%
Ec-MreB DLSIDLGTANT EATGSMVVDIGGGTVAV RGMVLTGGGALL 15.6%
Ec-ParM LVFIDDGSTNI ELDSLLIIDLGGTTISQ THVMVIGGGAEL 7.8%
Ec-FtsA VVGLEIGTAKV RELGVCVVDGIGGTIAV AGIVLTGGAAQI 9.4%
(c)
Wild type
ΔvolA*
ns
Motility halo diameter (mm)
35
30
25
20
Wild type
ΔvolA*
(d)
*
Aspect ratio
10
7.5
5
2.5
0
Wild type
volA::volA-msfGFP
(e)
Wild type + pTA963
ΔvolA* + pTA963
ΔvolA* + volA
**
****
****
Aspect Ratio
15
10
5
0
Wild type + pTA963
ΔvolA* + pTA963
ΔvolA* + volA
(f)
Rods and disks
Early log
Mid log
****
***
***
Mean fluorescence per cell
60
40
20
0
8
6
4
2
0
Early log
Mid log
Rods
Disks
Rods
Disks
Early log
Mid log
Aspect Ratio
5
4
3
2
Rods
Disks
0
2,000
4,000
6,000
Mean fluorescence per cell

Supplementary Figure 8: Volactin structural comparison, ΔvolA* *motility and complementation, and quantification of volactin fluorescence in rods and disks between early log and mid log.* **(a)** Structural comparison of actin homologs. From left to right, crystallographic structures of *Drosophila melanogaster* [4], *Caulobacter crescentus* [5], and *Pyrobaculum calidifontis* [6] actins and a structural model for *Hfx. volcanii* volactin as predicted by AlphaFold 2. **(b)** Sequence comparison of nucleotide binding sites of *Hfx. volcanii* volactin, *D. melanogaster* actin, *E. coli* MreB, *E. coli* ParM, and *E. coli* FtsA. **(c)** Left and middle panels, motility halo images for wild type and Δ*volA**. Colonies were stab-inoculated into 0.35% agar and incubated for two days at 45°C and one day at room temperature. Image for each strain is representative of 12 biological replicates. Scale bars indicate 10 mm. Right panel, quantification of motility halo diameter. df = 22. Halo diameters were assessed using an unpaired, two-tailed t-test. $p = 0.2559$. 'ns' is not significant. **(d)** Aspect ratio comparison between wild type and the chromosomal replacement volA::volA-msfGFP strains at OD_{600} of 0.3. Cells were segmented and analyzed to a n = 4406 and 3702, respectively. *$p<0.05$. **(e)** Mid-log (OD_{600} between 0.3 and 0.315) shape images for wild type + empty vector pTA963, Δ*volA** + empty vector pTA963, and Δ*volA** + *volA*, each representative of three biological replicates, and quantification of cellular aspect ratio. n = 1960, 1507, and 1872 for wild type + empty vector, Δ*volA** + empty vector, and Δ*volA** + *volA*, respectively. Aspect ratio comparisons were assessed using an unpaired, nonparametric, two-tailed Kolmogorov-Smirnov test. ****$p<0.0001$, **$p=0.0086$. Effect size is 0.091 between wild type + pTA963 and Δ*volA** + *volA* and -0.334 between Δ*volA** + pTA963 and Δ*volA** + *volA*. Aspect ratios <2 are considered disks and/or short rods. Scale bars are 5 μm. **(f)** Plots showing the quantification of GFP-tagged volactin fluorescence in early log versus mid log, early-log rods versus disks, and mid-log rods versus disks as well as the correlation between volactin-msfGFP signal and aspect ratio.

****p=0.0005 (left), ***p=0.002 (middle), ***p=0.0015 (right). Boxplots show the mean (center line between boxes), interquartile range (boxes), 95% confidence interval of the mean (notch), as well as the lowest and highest 25th percentile of the distribution (whiskers). Source data are provided as a Source Data file.

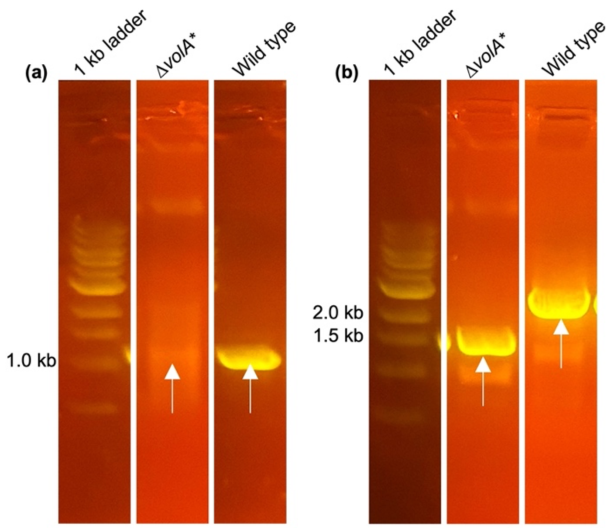

Supplementary Figure 9: Gel showing partial deletion for volA. **(a)** Gel from PCR of genomic DNA isolated from Δ*volA** using primers 2015_inside_fwd and 2015_inside_rvr revealed a faint band at approximately 1.0 kb (1038 bp gene size), as indicated by white arrows. **(b)** Gel from PCR of genomic DNA isolated from Δ*volA** using primers 2015_KO_up_fwd and 2015_KO_down_rvr revealed a band at approximately 1.5 kb (~750 bp upstream of gene + ~750 bp downstream of gene) for Δ*volA** and 2.5 kb (~750 bp upstream of gene + 1038 bp gene size + ~750 bp downstream of gene) for wild type as indicated by white arrows. All images shown are cut from the original images. Source data are provided as a Source Data file.

Supplementary Table 1: Plasmids, strains, and primers used to construct recombinant plasmids in this study.

**Name**	**Relevant characteristic(s)**	**Source**
Plasmids		
pTA131	Amp^r, pBluescript II with BamHI-XbaI fragments from pGB70 harboring *pfdx-pyrE2*	[7]
pTA963	Amp^r, *pyrE2* and *hdrB* markers, Trp-inducible (p.*tna*) promoter	[8]
pAD12	pTA131 carrying fragment with ~1500 nucleotides of fused upstream and downstream regions of *hvo_B0194*	This study
pHS1	pTA131 carrying fragment with ~1500 nucleotides of fused upstream and downstream regions of *rdfA*	This study
pHS2	pTA131 carrying fragment with ~1500 nucleotides of fused upstream and downstream regions of *sph3*	This study
pHS3	pTA131 carrying fragment with ~1500 nucleotides of fused upstream and downstream regions of *ddfA*	This study
pAD14	pTA131 carrying fragment with ~1500 nucleotides of fused upstream and downstream regions of *volA*	This study
pHS4	pTA963 carrying *rdfA*	This study
pHS5	pTA963 carrying *sph3*	This study
pHS6	pTA963 carrying *ddfA* as originally annotated	This study
pHS7	pTA963 carrying extended *ddfA* (*ddfA^{ext}*), based on the updated annotation	This study
pHS8	pTA963 carrying *volA*	This study
E. coli strains		

DH5α	F⁻ ϕ80Δ*lacZ*ΔM15 (*lacZYA-argF*)*U169 recA1 endA1 hsdR17*(r_K^- m_K^-) *phoA supE44 thi-1 gyrA96 relA1*	Invitrogen
DL739	MC4100 *recA dam-13*::Tn*9*	[9]
Hfx. volcanii strains		
H53	Δ*pyrE2* Δ*trpA*	[7]
H98	Δ*pyrE2* Δ*hdrB*	[7]
H295	H295 (Δ*pyrE2* Δ*trpA* Δ*rad50* Δ*mre11 bga-Ha-Kp*)	[10]
H26	Δ*pyrE2*	[7]
Δ*cetZ1*	H98 Δ*cetZ1*	[11]
JK3	H295 *hvo_B0194::tn* (secondary mutation in *hvo_1212*)	This study
JK5	H295 *hvo_B0364::tn* (secondary mutation in *hvo_1212*)	This study
SAH1	H295 *hvo_2176::tn*	[12]
AD12	H53 Δ*hvo_B0194*	This study
HS37	H53 Δ*rdfA* (pTA131 plasmid still integrated)	This study
HS50	H53 Δ*rdfA* (only used for complementation and growth curve; shape and motility phenotypes same as for HS37)	This study
HS211	H53 Δ*sph3*	This study
HS42	H53 Δ*ddfA*	This study
HS45	H53 Δ*volA** (partial)	This study
HS40	H53 containing empty vector pTA963	This study
HS204	HS50 containing empty vector pTA963	This study
HS51	HS50 containing pHS4	This study
HS213	HS211 containing empty vector pTA963	This study

HS215	HS211 containing pHS5	This study
HS43	HS42 containing empty vector pTA963	This study
HS44	HS42 containing pHS6	This study
HS215	HS42 containing pHS7	This study
HS52	HS45 containing empty vector pTA963	This study
HS53	HS45 containing pHS8	This study
aBL126	H26 *volA*::*volA*-40aa-msfGFP-pyrE2	This study
aBL170	H26 *volA*::*volA*-40aa-msfGFP-pyrE2 ftsZ1::ftsZ1-EG-mApple-I-mevR	This study
Primers	5' to 3' sequence	
B0194_KO_up_fwd	ctagctcgaggtccgcttgatatccgac	This study
B0194_KO_up_rvr	tcgcccgtttactggaagcactctggc	This study
B0194_KO_down_fwd	tgcttccagtaaacgggcgagtttctg	This study
B0194_KO_down_rvr	gatctctagagataagccagtcgaggacg	This study
2174_KO_up_fwd	attatctagagcttgtggttccgtacgag	This study
2174_KO_up_rvr	gttggtgagcccgaatcgtgtgacttcgccggcg	This study
2174_KO_down_fwd	cgccggcgaagtcacacgattcgggctcaccaac	This study
2174_KO_down_rvr	aatactcgagcgtctaattcggcttggtgc	This study
2175_KO_up_fwd	attattctagatcacgtctgataatccatgggt	This study
2175_KO_up_rvr	gctgtcagggcacgtcaggtgtcggatgtggatgtgtt	This study
2175_KO_down_fwd	aacacatccacatccgacacctgacgtgccctgacagc	This study
2175_KO_down_rvr	aatactcgaggagggcgagcggtcc	This study
2176_KO_up_fwd	attatctagaccacatcgtccgcgcg	This study
2176_KO_up_rvr	cgaacgacccggcagaggctgcggcgaccgc	This study
2176_KO_down_fwd	gcggtcgccgcagcctctgccgggtcgttcg	This study
2176_KO_down_rvr	attactcgaggagctggatactgctcgttatc	This study

2015_KO_up_fwd	ctagctcgagccagttcgtcggcgcgtatc	This study
2015_KO_up_rvr	ggaccggcgcacctcacgcgtcgtaggg	This study
2015_KO_down_fwd	cgcgtgaggtgcgccggtccgagctgac	This study
2015_KO_down_rvr	gatctctagacctgccacaccgagtcgaac	This study
2174_OE_fwd	tatattccatatgatgcgagactctggaaggtcgg	This study
2174_OE_rvr	tatattgaattcctagtgatggtgatggtgatgcgggccg ccctcgtctcgctcgcgt	This study
2175_OE_fwd	tatattcatatgatgaacgattcagaggcacttcaggcg gc	This study
2175_OE_rvr	tatattgaattctcagtgatggtgatggtgatgcgggccg ccggcggccggcgtcacgtcggtg	This study
2176_OE_fwd	tatattccatatggtggacaaacacgcccgcg	This study
2176_OE_fwd_newannot	tatattcatatgatgggacgatactggggggacg	This study
2176_OE_rvr	tatattggatccttagtgatggtgatggtgatgcgggccg ccccggcccgcgtcc	This study
2015_OE_fwd	atattccatatgatggcgaaaggccttgacgta	This study
2015_OE_rvr	tatattgaattctcagtgatggtgatggtgatgcgggccg ccgttc	This study
B0194_inside_fwd	actccgactcaaccgacgca	This study
B0194_inside_rvr	gacgaggcggtccgtctcga	This study
2174_inside_fwd	atgcgagactctggaaggtc	This study
2174_inside_rvr	ctactcgtctcgctcgcg	This study
2175_inside_fwd	atgaacgattcagaggcactt	This study
2176_inside_fwd	gtggacaaacacgcccg	This study
2176_inside_rvr	ttaccggcccgcgtc	This study
2015_inside_fwd	atggcgaaaggccttgacgtagg	This study
2015_inside_rvr	tcagttcgattccggcgcgg	This study
oBL174	gacgaggaagacgaggagg	This study

oBL175	catcctgactcgcgg	This study
oBL354	cttgagggtagcggac	This study
oBL24	ccatcccctcccatgtcatttgtaaagttcatccattccat g	This study
oHV6	ttagccgtcggcgtc	[13]
oHV7	cgtggataaaacccctcg	[13]
oBL232	cgtagatgcggttgagatg	This study
oBL248	cgaggggttttatccacggcgccggtccgagctga	This study
oHV3	cgtcctccgtaaaccg	This study
oHV4	gtccgctaccctcaagctcgacgtagtcgatgtct	This study
oHV140	gggaggtgacgcctgatggtctcgaagggcg	This study
oHV170	ccatcccctcccatgtcacttgtagagctcgtcc	This study
oBL36	cgaggaagcggaaga	This study
oBL37	catgggaggggatgg	This study
oBL308	ggatctaaatcaaaagaatagaccgatcgagccgtccc g	This study
oHV171	tcttccgcttcctcgtcgagccgtcccg	This study

Supplementary Table 2: Homologs to RdfA and Sph3 (SMC-like protein). Proteins belong to the same group when they show more than 55% protein sequence identity. Only sequences from the genomes under continuous survey are clustered into groups. In several cases, only the SMC-like protein met the cutoff for grouping. The following organisms are under continuous annotation survey: i) three species from *Haloferax* (HVO, *Hfx. volcanii*; HFX, *Hfx. mediterranei*; HfgLR, *Hfx. gibbonsii*); ii) *Haloquadratum walsbyi* (Hqrw); iii) two species from *Haloarcula* (rrnAC, rrnB and pNG, *Har. marismortui*; HAH, *Har. hispanica*); iv) two species from *Natronomonas* (NP, *Nmn. pharaonis*; Nmlp, *Nmn. moolapensis*); v) *Natrialba magadii* (Nmag); vi) two species from *Halobacterium* (OE, *Hbt. salinarum*; Hhub, *Hbt. hubeiense*); vii) *Halohasta litchfieldiae* (halTADL). The following genomes from 12 additional genera were analyzed: *Halalkalicoccus jeotgali* (HacjB3), *Halogeometricum borinquese* (Hbor), *Halomicrobium mukahataei* (Hmuk), *Halopiger xanaduensis* (Halxa), *Halorhabdus tiamatea* (HTIA), *Halorubrum lacusprofundi* (Hlac), *Haloterrigena turkmenica* (Htur), *Halovivax ruber* (Halru), *Natrinema pellirubrum* (Natpe), *Natronobacterium gregoryi* (Natgr), *Natronococcus occultus* (Natoc), *Salinarchaeum sp.* Harcht-Bsk1 (L593), halophilic archaeon DL31 (Halar). Serial, divergent, and convergent refer to the gene neighborhood configuration, i.e. the direction of transcription of one gene relative to the other. If the configuration is serial or divergent, positive numbers indicate the gene distance; negative numbers indicate a short gene overlap. If the configuration is serial, R-S or S-R refer to the order of the RdfA family (R) or SMC-like (S) encoding genes.

Group	Species to which protein belongs	RdfA family	SMC-like protein	Synteny	Comment
1	*Haloferax volcanii*	HVO_2174 (RdfA)	HVO_2175 (Sph3)	adjacent	serial-R-S, +35

2	*Haloferax volcanii*	HVO_A0179	HVO_A0180	adjacent	serial-R-S, -4
3	*Haloferax volcanii*	HVO_B0117	HVO_B0118	adjacent	serial-R-S, +75
4	*Haloferax volcanii*	HVO_B0174	HVO_B0173	adjacent	serial-S-R, +360
1	*Haloferax mediterranei*	HFX_2233	HFX_2234	adjacent	serial-R-S, +26
5	*Haloferax mediterranei*	HFX_6157	HFX_6156	adjacent	divergent, +102
1	*Haloferax gibbonsii*	HfgLR_12455	HfgLR_12450	adjacent	serial-R-S, +34
3	*Haloferax gibbonsii*	HfgLR_20520	HfgLR_20525	adjacent	serial-R-S, +79
6	*Haloquadratum walsbyi*	Hqrw_1956	Hqrw_1957	adjacent	serial-S-R, +217
7	*Haloquadratum walsbyi*	Hqrw_2741	-	-	
6	*Haloarcula marismortui*	rrnAC2249	rrnAC2250	adjacent	serial-S-R, +44
8	*Haloarcula marismortui*	rrnB0193	-	-	
9	*Haloarcula marismortui*	pNG7224	pNG7225	adjacent	serial-R-S, -4
10	*Haloarcula marismortui*	pNG7385	pNG7386	adjacent	convergent
6	*Haloarcula hispanica*	HAH_2718	HAH_2719	adjacent	serial-S-R, +44
8	*Haloarcula hispanica*	HAH_4309	-	-	

9	*Haloarcula hispanica*	HAH_5326	HAH_5325	adjacent	serial-R-S, -4
10	*Haloarcula hispanica*	HAH_5179	HAH_5178	adjacent	convergent
11	*Natronomonas pharaonis*	NP_3408A	NP_3410A	adjacent	serial-R-S, -4
12	*Natronomonas pharaonis*	NP_4260A	NP_4262A	adjacent	serial-R-S, +136
12	*Natronomonas moolapensis*	Nmlp_3023	Nmlp_3024	adjacent	divergent, +297
13	*Natrialba magadii*	Nmag_0804	-	-	
14	*Natrialba magadii*	Nmag_2907	Nmag_2908	adjacent	convergent
15	*Halobacterium salinarum*	OE_5048F	OE_5049F	adjacent	serial-R-S, +9
16	*Halobacterium salinarum*	OE_5211F	OE_5212F	adjacent	serial-R-S, +104
17	*Halobacterium hubeiense*	Hhub_2395	Hhub_2396	adjacent	serial-R-S, +51
18	*Halobacterium hubeiense*	Hhub_2448	-	-	
19	*Halohasta litchfieldiae*	halTADL_0031	halTADL_0032	adjacent	serial-R-S, +44
9	*Halohasta litchfieldiae*	halTADL_2636	halTADL_2637	adjacent	serial-R-S, +2
20	*Halohasta litchfieldiae*	halTADL_2656	halTADL_2657	adjacent	serial-R-S, +137
17	*Halorubrum lacusprofundi*	Hlac_2235	Hlac_2244	vicinity	separated by eight genes

18	*Halorubrum lacusprofundi*	Hlac_2591	-	-	
-	*Halorubrum lacusprofundi*	-	Hlac_2789	-	
1	*Halogeometricum boringuese*	Hbor_31810	Hbor_31800	adjacent	serial-R-S, +51
3	*Halogeometricum borinquese*	Hbor_36760	Hbor_36770	adjacent	serial-R-S, +137
13	*Haloterrigena turkmenica*	Htur_0821	Htur_0820	adjacent	serial-R-S, +91
9	*Haloterrigena turkmenica*	Htur_3953	Htur_3954	adjacent	serial-R-S, +2
-	*Haloterrigena turkmenica*	-	Htur_4125	-	
12	*Haloterrigena turkmenica*	Htur_4415	Htur_4414	adjacent	serial-R-S, +87
-	*Haloterrigena turkmenica*	Htur_4715	Htur_4714	adjacent	serial-R-S, +203
-	*Halomicrobium mukahataei*	Hmuk_0119	-	-	
-	*Halomicrobium mukahataei*	Hmuk_2049	Hmuk_2048	adjacent	convergent
-	Halophilic archaeon DL31	Halar_0328	Halar_0329	adjacent	serial-R-S, +275
-	Halophilic archaeon DL31	Halar_1101	Halar_1102	adjacent	serial-R-S, +87
-	Halophilic archaeon DL31	Halar_1116	-	-	
-	*Halopiger xanaduensis*	Halxa_0987	-	-	

-	*Halopiger xanaduensis*	-	Halxa_1877	-	
13	*Halopiger xanaduensis*	Halxa_2173		-	
17	*Natrinema pellirubrum*	Natpe_1605	Natpe_1604	adjacent	serial-R-S, +61
15	*Natrinema pellirubrum*	Natpe_1870	Natpe_1871	adjacent	serial-R-S, +50
-	*Natrinema pellirubrum*	Natpe_4419	Natpe_4420	adjacent	serial-R-S, +2
14	*Natronobacterium gregoryi*	-	Natgr_1168	-	
13	*Natronobacterium gregoryi*	Natgr_3161	-	-	
13	*Natronococcus occultus*	Natoc_0345	Natoc_0346	adjacent	serial-R-S, +66
-	*Natronococcus occultus*	-	Natoc_4085	-	
-	*Halalkalicoccus jeotgali*	HacjB3_09675	HacjB3_09670	adjacent	serial-R-S, +50
-	*Halalkalicoccus jeotgali*	-	HacjB3_17161	-	
-	*Halalkalicoccus jeotgali*	-	HacjB3_17473	-	
-	*Halorhabdus tiamatea*	HTIA_1062	HTIA_1063	adjacent	serial-R-S, +44
-	*Halorhabdus tiamatea*	HTIA_p2913	HTIA_p2914	adjacent	serial-R-S, +46
-	*Salinarchaeum sp.* Harcht-Bsk1	L593_06415	L593_06420	adjacent	serial-R-S, +69

Supplementary Table 3: Parameters for the mass spectrometric analysis of cell shape samples. A Q Exactive™ HF mass spectrometer (Thermo Fisher Scientific) was employed to measure samples for the comparison between H53, H98, Δ*cetZ1*, and JK3. An Orbitrap Eclipse™ mass spectrometer (Thermo Fisher Scientific) was employed to measure samples for the comparison between H53, Δ*ddfA*, and Δ*rdfA*.

	Q Exactive™ HF	Orbitrap Eclipse™
Chromatography		
Column	nanoEase M/Z Peptide BEH C18 column, Waters Corporation, 1.7 um particle size, 75 um x 250 mm	
Column oven	50°C	
Flow rate	300 nl/min	
Buffer system	Buffer A: 0.1% formic acid in H_2O<br>Buffer B: 0.1% formic acid in acetonitrile	
Gradient	1 min 3% B;<br>increase to 17% B over 39 min;<br>increase to 32% B over 20 min;<br>increase to 90% B over 2 min;<br>9 min 90% B;<br>decrease to 3% B over 2 min;<br>17 min 34% B	
Mass spectrometry, general settings		
Ion mode	positive	
Excluded charge states	1, >6, unknown	
Dynamic exclusion	10 s	
MS1 settings		
Resolution	60,000	120,000
Maximum injection time	100 ms	50 ms
AGC target	1e6	250% (1e6)

Detector	Orbitrap	
Scan range	400-2000 *m/z*	
MS2 settings		
Resolution	15,000	
Detector	Orbitrap	
AGC target	1e5	200% (1e5)
Maximum injection time	200 ms	dynamic
Data-dependent acquisition	TopN with N=20	cycle time 3 s
Scan range	fixed first 130 *m/z*	automatic
Fragmentation	Higher-energy collisional dissociation	
Normalized collision energy	stepped: 25, 30, 35	

Supplementary Note 1: Limitations of using genetic screens for identification of shape- and motility-related genes.

Transposon mutant screens for hypermotile mutants allowed for the isolation of mutant strains with shape defects, thus enabling identification of genes potentially important for both motility and shape. However, results from these screens are inherently limited to genes that affect both motility and shape and are non-essential, having, at most, minor effects on growth rates. These limitations of genetic screens prevent their use for more comprehensive analyses of shape transition pathways and, together with their laborious nature, render genetic screens less suitable for a more comprehensive identification of proteins involved in cell-shape pathways.

Supplementary Note 2: Strain descriptions.

Strain H53 (Δ*pyrE2* Δ*trpA*) is used as the parent strain for all clean gene deletions and is referred to as "wild type" throughout this manuscript. Strain H53 is derived from strain H26 (Δ*pyrE2*) [7]. The Δ*cetZ1* mutant is based on parent strain H98 (Δ*pyrE2* Δ*hdrB*), which is also derived from strain H26 [7]. The additional selection marker *hdrB* is expected to be neutral with respect to cell shape.

Strains JK3, JK5, and SAH1 are mutants of the transposon insertion library [14]. The background of this library is strain H295 (Δ*pyrE2* Δ*trpA* Δ*rad50* Δ*mre11 bga-Ha-Kp*) [10]. The genealogy of strain H295 is strain H204 (Δ*pyrE2* Δ*rad50* Δ*mre11 bga-Ha-Kp*), strain H115 (Δ*pyrE2 bga-Ha-Kp*), strain H54 (Δ*pyrE2 bga-Ha*), strain H26 [10].

It should be noted that the Δ*trpA* mutation of strain H295 was complemented during generation of the transposon insertion library, as the transposon constructs carry the *trpA* gene as a selection marker. The replacement of the *bgaH* gene of *Hfx. volcanii* by a mutant version of the

gene from *Haloferax alicantei* was made to allow for assays related to *rad50* and *mre11* [10]. It is highly unlikely that this mutation has any shape effect. Usage of a strain mutated in Δ*rad50* and Δ*mre11* had been considered helpful for generation of a transposon insertion library. These mutations are also likely to be neutral with respect to cell shape.

Thus, even though distinct parents have been used for the mutants, all of them differ by only a few genes, as they are all derived from the same ancestral strain H26 (Δ*pyrE2*) [7]. The genealogy of strain H26 has been recently described [12]. Strain H26 is based on strain DS70 [15], which is a direct descendent of wild-type strain DS2^T [16,17]. To generate strain DS70, the wild-type strain was cured of the small plasmid pHV2 [15]. During curing, plasmid pHV4 has been inadvertently integrated into the chromosome [18].

Supplementary Note 3: Clustering analysis for quantitative proteomics results of strains ΔrdfA*,* ΔddfA*, and wild type.*

The variance-sensitive clustering analysis (Supplementary Fig. 5b-d; Supplementary Fig. 6; Supplementary Data 2) showed protein abundance patterns for each condition and strain and thus enabled identification of specific clusters with proteins that may serve similar functions or may be part of the same pathway. For six of the fourteen clusters, differences in abundance were mostly due to changes in growth phase, with only minor differences between the strains. They could be grouped into clusters of proteins with higher abundance in late log (clusters 1, 8, and 10; Supplementary Fig. 6) or higher abundance in early log (cluster 2, 3, and 4; Supplementary Fig. 6). Proteins in these clusters were likely involved in cellular processes that correspond to the respective growth phase but were unlikely to be important for shape.

In contrast, the protein abundance patterns of clusters 11, 12, and 14 indicated that the

corresponding proteins were involved in cell-shape determination. Besides their likely importance for cell-shape determination as discussed in the main results, the proteins present in shape-specific clusters also revealed potential connections between shape and adhesion. Two proteins in cluster 11, GdhA1 (HVO_1451), a glutamate dehydrogenase, and hypothetical protein HVO_2447, have been shown before to likely be involved in surface adhesion, as transposon insertions in the genes of either protein result in adhesion defects [19]. Thus, the regulation of cell-shape transitions may be important for cellular adhesion to surfaces. Moreover, zinc-finger protein HVO_0758 was present in cluster 12, and previous work has shown that deletion of *hvo_0758* results in a loss in swarming ability along with an increase in biofilm formation relative to wild type [20], the latter of which aligns with the fact that genes potentially important for disk formation result in a defect in adhesion when absent.

While cluster 6 showed an overall similar abundance pattern as cluster 11, it was less pronounced, with only slightly higher abundances in early log for proteins from Δ*rdfA* compared to wild type and Δ*ddfA* (Supplementary Fig. 6). The roles of these proteins in disk-shaped cells therefore remain to be elucidated. The protein abundance patterns for the remaining four clusters (5, 7, 9, and 13) showed differences for both shape mutants compared to wild type (Supplementary Fig. 6). Since both mutants have distinct shapes, it is unlikely that these clusters correspond to proteins involved in shape-specific processes. Instead, they might be part of stress responses as a result of their altered shapes.

Supplementary Note 4: Genomic regions likely involved in cell-shape determination as indicated by comparative quantitative proteomics.

Further analysis of the proteins that showed a likely importance in shape determination

based on the knowledge-based filtering using comparisons between wild type and shape mutants (Supplementary Fig. 7; Supplementary Data 1 and 3) revealed several genomic regions that encode for multiple proteins likely involved in shape determination. The importance of the genomic region between *hvo_2160* and *hvo_2176* (*ddfA*) was indicated by the performed genetic screens as well as the proteomic comparisons including JK3 and Δ*cetZ1*, and additional genes were suggested through the proteomic analysis of Δ*rdfA* and Δ*ddfA*. The glycoproteins HVO_2160 and HVO_2161, the ABC transporter HVO_2163, and the potential AAA-type ATPase MoxR (HVO_2168), showed higher abundances in disks, while RdfA (HVO_2174) and Sph3 (HVO_2175) were more abundant in rods. While no quantitative proteomics data could be obtained for DdfA, potentially due its small size, its deletion resulted in a rod-only phenotype (see main results).

Another genomic region encoding for proteins with potential roles in cell shape is that which covers various enzymes of the Agl15-dependent *N*-glycosylation pathway: Agl5, Agl7, Agl9, Agl10, Agl11, Agl12, and Agl14. All of these were significantly more abundant in disks than rods (Supplementary Fig. 7). While none of them was part of a cluster, their abundance patterns were similar to clusters 6 and 11 (Supplementary Data 2), suggesting importance for disk formation. Two *N*-glycosylation pathways exist in *Hfx. volcanii*: AglB-dependent and Agl15-dependent *N*-glycosylation, and deletion mutants of *aglB* or *agl15* have been shown to have shape defects [21]. Specifically, while Δ*agl15* makes rods longer into mid- and late-log growth phases than wild type, suggesting the importance of the Agl15-dependent *N*-glycosylation pathway in disk formation, Δ*aglB* makes more disks in early log than wild type, implicating the AglB-dependent *N*-glycosylation pathway in rod formation [21]. Our results, in combination with previous work, suggest that different shapes may be associated with distinct *N*-glycans. Specifically, Agl15-

dependent *N*-glycosylation seems to be involved in the transition to disks, but its exact role remains to be elucidated. It is possible that specific *N*-glycans allow for cellular processes to occur that are specific to certain shapes, such as motility, adhesion, or nutrient acquisition. However, it is important to note that *N*-glycosylation pathways might not be direct effectors of cell-shape transition; rather, any changes in type or extent of *N*-glycosylation might be an adaptation which occurs after the transition and thus may be downstream of the actual shape change. In a separate analysis, we identified and quantified multiple *N*-glycoproteins. However, the significant protein abundance differences (PEP<0.05) in shape-relevant comparisons were only observed for *N*-glycosylated ArlA1, ArlA2, as well as HVO_2160. The abundance ratios for the corresponding *N*-glycopeptides were similar to the non-glycosylated peptides of the same protein; thus, a specific effect on the degree of *N*-glycosylation could not be concluded. It should be noted that all reliably quantified *N*-glycopeptides were of the AglB-dependent type, and enzymes of the AglB-dependent glycosylation pathway were not observed in shape-related clusters or in lists of candidate proteins after knowledge-driven filtering. Conversely, enzymes of the Agl15-dependent *N*-glycosylation pathway were strong candidates to be involved in shape, but only a handful of *N*-glycopeptides with Agl15-dependent modifications were identified to date [21], and none of them could be reliably quantified here.

Lastly, the third region between *hvo_2013* and *hvo_2020* encodes for proteins CetZ5 (HVO_2013) and VolA (HVO_2015) along with uncharacterized proteins HVO_2016, HVO_2018, and HVO_2020, all of which showed higher abundance in disks (Supplementary Fig. 7). This genomic region was particularly interesting, since VolA was also part of the five proteins that showed significant abundance differences across all shape-specific comparisons, not only for those including Δ*rdfA* and Δ*ddfA* but also for JK3 and Δ*cetZ1*.

Supplementary Note 5: Identification and characterization of volactin.

Volactin was originally identified by BLAST searching the *Hfx. volcanii* genome with annotated actin homolog sequences from various archaeal and bacterial species. The *Halapricum salinum* FtsA-like protein, WP_049993655.1 [https://www.ncbi.nlm.nih.gov/protein/WP_049993655.1], retrieved WP_004043705.1 [https://www.ncbi.nlm.nih.gov/protein/WP_004043705.1] entered as "hypothetical protein" in the *Hfx. volcanii* genome. Using SwissModel to fit the sequence to the crystallographic structure of MreB from *Thermotoga maritima* confirmed that the *Hfx. volcanii* sequence was compatible with an actin-like fold.

To further investigate HVO_2015 (volactin), as it was selected as a strong candidate in both rounds of proteomics, we explored its predicted AlphaFold structure [22] from the EMBL-EBI Database (https://alphafold.ebi.ac.uk/entry/D4GU28). Using the Dali server [23] to seek for possible homologs, we obtained a vast majority of actin-related proteins, with the top hit being the bacterial rod shape-determining MreB [24]. This finding is in line with results from our previous BLAST search. We compared the predicted structure of HVO_2015 with experimentally solved actin structures across eukaryotic and prokaryotic species (Supplementary Fig. 8a), revealing strong similarities in their domain organization. Additionally, HVO_2015 sequence alignment with eukaryotic and bacterial actin families showed conserved ATP-binding motifs that were previously characterized [25], specifically Phosphate 1 (xhxh**D/E**xGpxx), Phosphate 2 (xssxhhh**D**x**G**ssshxx), and Adenosine (xxhhhx**GG**ssxx) (Supplementary Fig. 8b).

Analyzing images of cells expressing volactin-msfGFP at early- (primarily rods) and mid-log (mixed rods and disks) growth phases, we observed volactin filaments in both populations

(Fig. 5e). However, a higher fluorescence signal from volactin-msfGFP polymers was observed in mid-log (μ=855±322) cells compared to early-log (μ=169±65) cells (Supplementary Fig. 8f). To determine whether this difference is related to growth phase or disk enrichment in the mid-log population, we used machine learning-based image segmentation and cellular aspect ratio to distinguish rods from disks [13]. Supporting our proteomics data, we concluded that disks exhibited approximately a 2-fold increase in volactin-msfGFP signal compared to rods in early-log (μ=340±161 and μ=166±58, respectively) and a 3-fold increase in mid-log (μ=2023±643 and μ=764±314, respectively) growth phases (Supplementary Fig. 8f). Interestingly, plotting the correlation between volactin-msfGFP signal and aspect ratio showed a skewed sub-population of rods with more volactin polymer material at mid- compared to early-log growth phase (Supplementary Fig. 8f).

<u>Supplementary References</u>

1. Abdul Halim, M. F. *et al.* Permuting the PGF Signature Motif Blocks both Archaeosortase-Dependent C-Terminal Cleavage and Prenyl Lipid Attachment for the Haloferax volcanii S-Layer Glycoprotein. *J. Bacteriol.* **198**, 808–815 (2016).
2. Babski, J. *et al.* Genome-wide identification of transcriptional start sites in the haloarchaeon Haloferax volcanii based on differential RNA-Seq (dRNA-Seq). *BMC Genomics* **17**, 629 (2016).
3. Hadjeras, L. *et al.* Revealing the small proteome of *Haloferax volcanii* by combining ribosome profiling and small-protein optimized mass spectrometry. *microLife* **4**, uqad001 (2023).
4. Rould, M. A., Wan, Q., Joel, P. B., Lowey, S. & Trybus, K. M. Crystal Structures of

Expressed Non-polymerizable Monomeric Actin in the ADP and ATP States. *J. Biol. Chem.* **281**, 31909–31919 (2006).

5. van den Ent, F., Izoré, T., Bharat, T. A., Johnson, C. M. & Löwe, J. Bacterial actin MreB forms antiparallel double filaments. *eLife* **3**, e02634 (2014).
6. Izoré, T., Kureisaite-Ciziene, D., McLaughlin, S. H. & Löwe, J. Crenactin forms actin-like double helical filaments regulated by arcadin-2. *eLife* **5**, e21600 (2016).
7. Allers, T., Ngo, H.-P., Mevarech, M. & Lloyd, R. G. Development of Additional Selectable Markers for the Halophilic Archaeon Haloferax volcanii Based on the leuB and trpA Genes. *Appl. Environ. Microbiol.* **70**, 943–953 (2004).
8. Allers, T., Barak, S., Liddell, S., Wardell, K. & Mevarech, M. Improved Strains and Plasmid Vectors for Conditional Overexpression of His-Tagged Proteins in Haloferax volcanii. *Appl. Environ. Microbiol.* **76**, 1759–1769 (2010).
9. Blyn, L. B., Braaten, B. A. & Low, D. A. Regulation of pap pilin phase variation by a mechanism involving differential dam methylation states. *EMBO J.* **9**, 4045–4054 (1990).
10. Delmas, S., Shunburne, L., Ngo, H.-P. & Allers, T. Mre11-Rad50 Promotes Rapid Repair of DNA Damage in the Polyploid Archaeon Haloferax volcanii by Restraining Homologous Recombination. *PLoS Genet.* **5**, e1000552 (2009).
11. Duggin, I. G. *et al.* CetZ tubulin-like proteins control archaeal cell shape. *Nature* **519**, 362–365 (2015).
12. Collins, M. *et al.* Mutations Affecting HVO_1357 or HVO_2248 Cause Hypermotility in Haloferax volcanii, Suggesting Roles in Motility Regulation. *Genes* **12**, 58 (2020).
13. Abdul-Halim, M. F. *et al.* Lipid Anchoring of Archaeosortase Substrates and Midcell Growth in Haloarchaea. **11**, 14 (2020).

14. Kiljunen, S. *et al.* Generation of comprehensive transposon insertion mutant library for the model archaeon, Haloferax volcanii, and its use for gene discovery. *BMC Biol.* **12**, 103 (2014).

15. Wendoloski, D., Ferrer, C. & Dyall-Smith, M. L. A new simvastatin (mevinolin)-resistance marker from Haloarcula hispanica and a new Haloferax volcanii strain cured of plasmid pHV2. *Microbiology* **147**, 959–964 (2001).

16. Mullakhanbhai, M. F. & Larsen, H. Halobacterium volcanii spec. nov., a Dead Sea halobacterium with a moderate salt requirement. *Arch. Microbiol.* **104**, 207–214 (1975).

17. Hartman, A. L. *et al.* The Complete Genome Sequence of Haloferax volcanii DS2, a Model Archaeon. *PLoS ONE* **5**, e9605 (2010).

18. Hawkins, M., Malla, S., Blythe, M. J., Nieduszynski, C. A. & Allers, T. Accelerated growth in the absence of DNA replication origins. *Nature* **503**, 544–547 (2013).

19. Legerme, G. *et al.* Screening of a Haloferax volcanii Transposon Library Reveals Novel Motility and Adhesion Mutants. *Life* **6**, 41 (2016).

20. Nagel, C., Machulla, A., Zahn, S. & Soppa, J. Several One-Domain Zinc Finger µ-Proteins of Haloferax Volcanii Are Important for Stress Adaptation, Biofilm Formation, and Swarming. *Genes* **10**, 361 (2019).

21. Schulze, S., Pfeiffer, F., Garcia, B. A. & Pohlschroder, M. Comprehensive glycoproteomics shines new light on the complexity and extent of glycosylation in archaea. *PLOS Biol.* **19**, e3001277 (2021).

22. Jumper, J. *et al.* Highly accurate protein structure prediction with AlphaFold. *Nature* **596**, 583–589 (2021).

23. Holm, L. Dali server: structural unification of protein families. *Nucleic Acids Res.* **50**,

W210–W215 (2022).

24. Garner, E. C. Toward a Mechanistic Understanding of Bacterial Rod Shape Formation and Regulation. *Annu. Rev. Cell Dev. Biol.* **37**, 1–21 (2021).

25. Bork, P., Sander, C. & Valencia, A. An ATPase domain common to prokaryotic cell cycle proteins, sugar kinases, actin, and hsp7O heat shock proteins. *Proc Natl Acad Sci USA* (1992).
