# Supplementary material for: Identification of structural and regulatory cell-shape determinants in Haloferax volcanii
Source: Nat Commun. 2024 Feb 15;15:1414. doi: 10.1038/s41467-024-45196-0 (PMC10869688; doi:10.1038/s41467-024-45196-0)
Supplement: Supplementary file 3 — Description of Additional Supplementary Files [file 41467_2024_45196_MOESM3_ESM.pdf]

Description of Additional Supplementary Files:

Supplementary Data 1: Proteins identified through proteomic comparisons with wild type, JK3, and Δ*cetZ1*. File includes all quantified proteins (tab 1), proteins that have significant abundance differences for at least one comparison (tab 2), and proteins that have significant abundance differences for wild type between early log and late log as well as at least one shape-specific comparison (tab 3).

Supplementary Data 2: Results of variance-sensitive clustering. All quantified proteins were clustered using VSClust, and the resulting cluster assignments are given together with the corresponding membership values. A membership value >0.5 was required for a protein to be assigned to a cluster.

Supplementary Data 3: Proteins identified through proteomic comparisons with wild type, Δ*rdfA*, and Δ*ddfA*. File includes all quantified proteins (tab 1), proteins that have significant abundance differences for at least one comparison (tab 2), proteins that have significant abundance differences for wild type between early log and late log as well as at least one shape-specific comparison (tab 3), and proteins that have significant abundance differences for wild type between early log and late log as well as both shape-specific comparisons (tab 4). Proteins that show significant abundance differences for wild type between early log and late log as well as both shape-specific comparisons in both proteomics experiments (JK3, Δ*cetZ1*, and wild type along with Δ*rdfA*, Δ*ddfA*, and wild type) are listed in tab 5.

Supplementary Movie 1: Volactin filament assembly and disassembly is independent of the division site. Colocalization of volactin- and FtsZ-tagged protein fluorescence reveals a lack of overlap between the two proteins, indicating spatial separation between volactin filament assembly and disassembly and cell division.

Supplementary Movie 2: Volactin filaments bind the membrane. 3-D super-resolution projections reveal volactin filaments span the cytoplasm and are bound from their tips to the membrane.
