# Supplementary material for: Identification of structural and regulatory cell-shape determinants in Haloferax volcanii
Source: Nat Commun. 2024 Feb 15;15:1414. doi: 10.1038/s41467-024-45196-0 (PMC10869688; doi:10.1038/s41467-024-45196-0)
Supplement: Supplementary file 9 — Reporting Summary [file 41467_2024_45196_MOESM9_ESM.pdf]

nature portfolio

Corresponding author(s): Mechthild Pohlschroder, Stefan Schulze, Alexandre Bisson-Filho

Last updated by author(s): Dec 5, 2023

Reporting Summary

Nature Portfolio wishes to improve the reproducibility of the work that we publish. This form provides structure for consistency and transparency in reporting. For further information on Nature Portfolio policies, see our Editorial Policies and the Editorial Policy Checklist.

Statistics

For all statistical analyses, confirm that the following items are present in the figure legend, table legend, main text, or Methods section.

n/a	Confirmed	
☐	☒	The exact sample size (*n*) for each experimental group/condition, given as a discrete number and unit of measurement
☐	☒	A statement on whether measurements were taken from distinct samples or whether the same sample was measured repeatedly
☐	☒	The statistical test(s) used AND whether they are one- or two-sided<br>*Only common tests should be described solely by name; describe more complex techniques in the Methods section.*
☒	☐	A description of all covariates tested
☒	☐	A description of any assumptions or corrections, such as tests of normality and adjustment for multiple comparisons
☐	☒	A full description of the statistical parameters including central tendency (e.g. means) or other basic estimates (e.g. regression coefficient) AND variation (e.g. standard deviation) or associated estimates of uncertainty (e.g. confidence intervals)
☐	☒	For null hypothesis testing, the test statistic (e.g. *F*, *t*, *r*) with confidence intervals, effect sizes, degrees of freedom and *P* value noted<br>*Give P values as exact values whenever suitable.*
☐	☒	For Bayesian analysis, information on the choice of priors and Markov chain Monte Carlo settings
☐	☒	For hierarchical and complex designs, identification of the appropriate level for tests and full reporting of outcomes
☒	☐	Estimates of effect sizes (e.g. Cohen's *d*, Pearson's *r*), indicating how they were calculated

Our web collection on statistics for biologists contains articles on many of the points above.

Software and code

Policy information about availability of computer code

Data collection	The optical density of the initial inoculum for the proteomics was measured using a BioTek PowerWaveX2 microplate spectrophotometer and BioTek Gen5 v.1.11.5 (Agilent).<br>Cell shape images were acquired using a Leica DFC9000 GT camera with Leica Application Suite X (version 3.6.0.20104) software.<br>Phase contrast images of cells and fluorescence of volactin-msfGFP filaments were recorded with a Hamamatsu ORCA Flash 4.0 v3 sCmos camera.<br>Growth curve data was obtained using a BioTek Epoch 2 microplate reader with BioTek Gen6 v.1.03.01 (Agilent).<br>3D super-resolution microscopy images were processed using NIS-Elements software (Nikon), using NIS.ai for denoising and 3D deconvolution.
Data analysis	The Python framework Ursgal (version 0.6.9) was used for all proteomic analyses. ThermoRawFileParser (version 1.1.2) and pymzML (version 2.5.0) were used for converting mass spectrometry raw files into mzML and MGF format, respectively. Protein database searches with MSFragger (version 3.0), X!Tandem (version vengeance), and MS-GF+ (version 2019.07.03) were performed against the theoretical proteome of Hfx. volcanii. Mass spectrometry runs were calibrated and search parameters were optimized using MSFragger. Peptide spectrum matches (PSMs) were statistically post-processed with Percolator (version 3.4.0). FlashLFQ (version 1.1.1) was used for label-free peptide and protein quantification. Log2-transformed fold changes were plotted as heatmaps using Plotly (https://plotly.com). Variance-sensitive fuzzy clustering was performed using VSClust (version 1.2). Cluster numbers were estimated using the minimal centroid distance and Xie-Beni index implemented in VSClust. All identification and quantification proteomics results, as well as the corresponding analysis scripts are made available through the Archaeal Proteome Project (http://archaealproteomeproject.org).<br>Brightfield shape images were quantified using CellProfiler (versions 4.2.1). Graphing of quantified of shape images was performed using GraphPad Prism version 9.3.1 and 10.0.2 for macOS (GraphPad Software, San Diego, California USA, www.graphpad.com). Effect size was calculated using PlotsOfDifferences.

Cell masks created from phase contrast images were generated through automated batch processing using Trackmate running on Cellpose2. From masks, cell aspect ratios were measured using Fiji. The fluorescence of volactin polymers was measured by first segmenting every structure with GFP signal using custom Fiji macros. The custom Fiji macros used in this study can be access through the Bisson Lab GitHub repository (https://github.com/Archaea-Lab/image-segmentation).
Motility halos were quantified using Fiji (ImageJ2) (version 2.3.0/1.53q). Statistical significance of halo diameters was assessed with an unpaired, two-tailed t-test using GraphPad Prism version 9.3.1 for macOS (GraphPad Software, San Diego, California USA, www.graphpad.com).
Syntenic clustering of rdfA and sph3 was performed against 120 haloarchaeal genomes with SyntTax (https://archaea.i2bc.paris-saclay.fr/SyntTax/). BLASTp was used to search for RdfA and Sph3 homologs.
dRNA-Seq and ribosome profiling data was obtained and then visualized with the Integrated Genome Browser.
3D super-resolution microscopy stacks were rendered into 3D images using Fiji.
Graphs for data visualization were plotted using PlotTwist and PlotsOfData. Cell shape images in Figures 2a and b, 4e, and 6 were generated using Clip Studio Paint Pro, version 1.10.2.

For manuscripts utilizing custom algorithms or software that are central to the research but not yet described in published literature, software must be made available to editors and reviewers. We strongly encourage code deposition in a community repository (e.g. GitHub). See the Nature Portfolio guidelines for submitting code & software for further information.

Data

Policy information about availability of data

All manuscripts must include a data availability statement. This statement should provide the following information, where applicable:

- Accession codes, unique identifiers, or web links for publicly available datasets
- A description of any restrictions on data availability
- For clinical datasets or third party data, please ensure that the statement adheres to our policy

The MS raw files generated for this study have been deposited to the ProteomeXchange Consortium (http://proteomecentral.proteomexchange.org) via the PRIDE partner repository 84 with the dataset identifier PXD040781, and all identification and quantification results have been included in the deposited dataset. Additional raw data for cell shape analyses and whole genome sequencing can be found on Zenodo (https://doi.org/10.5281/zenodo.8404691). Source data are provided with this paper, which includes cell shape quantification data for Figures 3 and 5 along with Supplementary Figures 1, 2, 4, and 8, growth curve data for Supplementary Figure 3, motility quantification data for Figure 3 along with Supplementary Figure 8, and knockout strain gels for Supplementary Figure 9. Additional data generated in this study that are provided in the Supplementary Information file include shape quantification data for ΔhvoB0194, ΔrdfA, Δsph3, ΔddfA, and ΔvolA* complementation strains, the growth curve for ΔrdfA, Δsph3, ΔddfA, and ΔvolA*, transcriptional start site data for ddfA, clustering estimation and graphs, proteomics heat map, actin structural comparisons, ΔvolA* motility data, and volactin fluorescence data. The strains described in this study can be obtained from the authors.
All scripts for the identification and quantification of peptides and proteins from MS datasets are made available through the Archaeal Proteome Project (http://archaealproteomeproject.org) and its associated GitHub repository (https://github.com/arcpp/ArcPP v1.4.0). The custom Fiji macros used in this study can be accessed through the Bisson Lab GitHub repository (https://github.com/Archaea-Lab/image-segmentation v1.0.0).

Research involving human participants, their data, or biological material

Policy information about studies with human participants or human data. See also policy information about sex, gender (identity/presentation), and sexual orientation and race, ethnicity and racism.

Reporting on sex and gender	N/A
Reporting on race, ethnicity, or other socially relevant groupings	N/A
Population characteristics	N/A
Recruitment	N/A
Ethics oversight	N/A

Note that full information on the approval of the study protocol must also be provided in the manuscript.

Field-specific reporting

Please select the one below that is the best fit for your research. If you are not sure, read the appropriate sections before making your selection.

[x] Life sciences [ ] Behavioural & social sciences [ ] Ecological, evolutionary & environmental sciences

For a reference copy of the document with all sections, see nature.com/documents/nr-reporting-summary-flat.pdf

Life sciences study design

All studies must disclose on these points even when the disclosure is negative.

Sample size: Sample sizes were chosen based on standards of the field for biological replicates and at least three biological replicates were performed as a minimum for statistical analyses. Sample sizes were deemed sufficient after statistical analyses revealed significant differences between the

	compared strains and conditions.
Data exclusions	No data was excluded from the analysis.
Replication	At least three biological replicates were analyzed per strain for the analyses described to ensure reproducibility. Results for all replicates are reported and were included in statistical analyses.
Randomization	The order of measurements for the proteomics samples was randomized.
Blinding	Blinding was not relevant for this study, because the study design as well as the largely automatized analysis of results precluded a significant impact of subjectivity on study outcomes.

Reporting for specific materials, systems and methods

We require information from authors about some types of materials, experimental systems and methods used in many studies. Here, indicate whether each material, system or method listed is relevant to your study. If you are not sure if a list item applies to your research, read the appropriate section before selecting a response.

Materials & experimental systems

n/a	Involved in the study
☒	☐ Antibodies
☒	☐ Eukaryotic cell lines
☒	☐ Palaeontology and archaeology
☒	☐ Animals and other organisms
☒	☐ Clinical data
☒	☐ Dual use research of concern
☒	☐ Plants

Methods

n/a	Involved in the study
☒	☐ ChIP-seq
☒	☐ Flow cytometry
☒	☐ MRI-based neuroimaging
